# Effects of Two Rg3-Enriched Korean Ginseng Preparations on Blood Pressure in Adults with Early-Stage Hypertension: A Randomized, Double-Blind, Placebo-Controlled Trial

**DOI:** 10.3390/nu18203448

**Published:** 2026-10-20

**Authors:** Jin-Hwan Oh, Yoo-Seok Cheong, Seong-Hoon Lim, Seong Lee, Jin-Hee Kim, Hyuna Jang, Youl Hour, Vladimir Vuksan, Hyang-Im Baek

**Affiliations:** 1 BTGin Co., Ltd., Daejeon 34024, Republic of Korea; jimhwano@naver.com (J.-H.O.); gin@btgin.com (J.-H.K.); heryoul@naver.com (Y.H.); 2 Department of Family Medicine, College of Medicine, Dankook University, Cheonan 31116, Republic of Korea; drloved@hanmail.net; 3 Department of Cardiology, Dankook University Hospital, Cheonan 31116, Republic of Korea; shlimd@gmail.com; 4 Research Institute of Clinical Medicine, Dankook University Hospital, Cheonan 31116, Republic of Korea; seong0909@gmail.com; 5 Department of Statistics, Sookmyung Women’s University, Seoul 04312, Republic of Korea; hyunaa@sookmyung.ac.kr; 6 Department of Nutritional Sciences, Temerty Faculty of Medicine, University of Toronto, Toronto, ON M5S 1A8, Canada; v.vuksan@utoronto.ca; 7 Department of Food Science & Nutrition, Woosuk University, Wanju 55338, Republic of Korea

**Keywords:** ginsenoside Rg3, *Panax ginseng*, Korean red ginseng, Korean white ginseng, blood pressure, functional food

## Abstract

**Background/Objectives**: Ginsenoside Rg3, a bioactive component of processed Korean ginseng, has demonstrated vasodilatory and blood pressure-lowering effects; however, clinical evidence on the long-term effects of Rg3-enriched ginseng preparations remains limited. This study evaluated the effects of two differently processed Rg3-enriched Korean ginseng preparations on blood pressure in adults with early-stage hypertension. **Methods**: In this 12-week, randomized, double-blind, placebo-controlled trial, 90 adults were randomly assigned to vitamin C-treated white ginseng (VTWG), enzyme-treated red ginseng (ETRG), or placebo (*n* = 30 per group). Both interventions provided approximately 40 mg/day of Rg3. Changes in systolic blood pressure (SBP) and diastolic blood pressure (DBP) were primary outcomes. **Results**: From baseline to week 12, SBP decreased by 7.27 ± 6.15 mmHg in the VTWG group and 9.86 ± 6.47 mmHg in the ETRG group, whereas it increased by 5.03 ± 6.52 mmHg in the placebo group (both adjusted *p* < 0.0001 vs. placebo). DBP decreased by 5.27 ± 5.58 and 6.32 ± 5.55 mmHg in the VTWG and ETRG groups, respectively, compared with a decrease of 0.90 ± 6.31 mmHg in the placebo group (adjusted *p* = 0.006, 0.0006, respectively). MAP also decreased significantly in both intervention groups compared with placebo (both adjusted *p* < 0.0001), whereas NO changes differed significantly only between ETRG and placebo (adjusted *p* = 0.017). No clinically meaningful safety concerns were identified. **Conclusions**: Twelve-week supplementation with either Rg3-enriched Korean ginseng preparation significantly reduced blood pressure compared with placebo, suggesting potential benefits for blood pressure management in adults with early-stage hypertension.

## 1. Introduction

Hypertension is a major modifiable risk factor for cardiovascular disease (CVD), including coronary heart disease, heart failure, and stroke [1,2,3]. The risk of cardiovascular events increases progressively with increasing blood pressure, and even modest elevations in blood pressure are associated with increased cardiovascular risk [2,3]. Importantly, the relationship between blood pressure and cardiovascular risk is continuous, highlighting the importance of blood pressure management from the early stages of blood pressure elevation [1,2,3]. Although pharmacological treatment is required for many individuals with hypertension, lifestyle modification and dietary strategies are also important components of blood pressure management [1]. Accordingly, there is continued interest in dietary approaches and food-derived bioactive compounds that may support blood pressure management, particularly in individuals with early-stage hypertension.

Among food-derived bioactive materials investigated for their cardiovascular effects, *Panax ginseng* C.A. Meyer has received considerable attention. Ginseng has a long history of use and is consumed in various processed forms, including white and red ginseng [4,5]. Ginsenosides, the major bioactive constituents of ginseng, are considered to contribute to many of its biological activities, including effects on vascular function [5,6]. Among individual ginsenosides, ginsenoside Rg3 has attracted particular attention because of its vasoactive properties [7,8,9,10]. Rg3 is formed during ginseng processing through the transformation of other major ginsenosides, and various processing techniques have been developed to increase its content [11]. These characteristics have led to increasing interest in Rg3-enriched ginseng preparations as a means of enhancing the potential vascular effects of ginseng.

The vascular effects of Rg3 are supported by several experimental studies. Nitric oxide (NO), produced in vascular endothelial cells through endothelial nitric oxide synthase (eNOS), plays an important role in the regulation of vascular tone [8,9]. Rg3 has been shown to promote vascular relaxation through mechanisms involving eNOS activation and NO production [7,10,12]. In addition, Rg3 has been reported to induce both endothelium-dependent and endothelium-independent vascular relaxation, suggesting that multiple mechanisms may contribute to its vasoactive effects [10,12]. Consistent with these findings, Korean red ginseng (KRG) has also been shown to influence blood pressure and NO production in experimental models [13].

Experimental studies using Rg3-enriched ginseng preparations provide further support for their potential blood pressure-related effects. Rg3-enriched KRG increased eNOS phosphorylation and NO production and improved endothelium-dependent vasorelaxation in spontaneously hypertensive rats [14,15]. In addition, Rg3-enriched KRG significantly reduced systolic blood pressure (SBP) and diastolic blood pressure (DBP), whereas conventional KRG did not significantly reduce blood pressure under the same experimental conditions [15]. An Rg3-enriched white ginseng preparation produced using vitamin C treatment also demonstrated blood pressure-lowering effects in spontaneously hypertensive rats [16]. These findings provide a biological rationale for increasing the Rg3 content of ginseng preparations to enhance their potential vascular and blood pressure-related effects.

Despite these experimental findings, clinical evidence regarding the effects of ginseng on blood pressure has not been entirely consistent. Clinical studies of conventional ginseng or KRG have reported variable effects on blood pressure and arterial stiffness in individuals with mild hypertension, hypertension, or metabolic syndrome [17,18,19]. A systematic review and meta-analysis of randomized controlled trials also suggested modest blood pressure-lowering effects of ginseng, although the available evidence was limited and heterogeneous [20]. Clinical evidence specifically evaluating Rg3-enriched ginseng preparations is even more limited. In a randomized, double-blind, crossover trial involving healthy young adults, a single dose of Rg3-enriched KRG significantly reduced central and brachial SBP and DBP, as well as mean arterial pressure, compared with control [21]. However, this study evaluated only acute responses following a single administration in healthy individuals. In a subsequent 12-week randomized controlled trial, co-administration of Rg3-enriched KRG and American ginseng improved blood pressure-related outcomes in individuals with type 2 diabetes and hypertension receiving standard medical therapy [22]. Additional vascular analyses from this trial showed a reduction in central SBP, although no significant changes were observed in several measures of endothelial function and arterial stiffness [23]. However, because Rg3-enriched KRG was administered together with American ginseng and the participants had concomitant type 2 diabetes and hypertension while receiving background pharmacotherapy, the independent long-term effects of Rg3-enriched Korean ginseng preparations remain unclear.

Accordingly, clinical evidence regarding the long-term effects of Rg3-enriched Korean ginseng preparations administered alone in individuals with early-stage hypertension remains limited. Therefore, this study aimed to evaluate the effects of 12-week supplementation with two differently processed Rg3-enriched Korean ginseng preparations, providing a comparable daily amount of Rg3, on blood pressure in adults with early-stage hypertension.

## 2. Materials and Methods

### 2.1. Ethics Statement

The study protocol was reviewed and approved by the Institutional Review Board (IRB) of Dankook University Hospital (DKUH IRB No. 2020-10-007; approved on 21 October 2020). The trial was retrospectively registered with the Clinical Research Information Service (CRIS) in the Republic of Korea on 26 May 2023 (registration number: KCT0008475). The delay in registration occurred because the investigators were not aware of the requirement for prospective clinical trial registration at the time of study initiation. All participants voluntarily provided written informed consent before participating in the study. The study was conducted in accordance with the principles of the Declaration of Helsinki and Good Clinical Practice guidelines.

### 2.2. Study Participants

Adults aged 20–70 years with early-stage hypertension were eligible for participation. In this study, early-stage hypertension was operationally defined as an SBP of 125–145 mmHg or a DBP of 80–95 mmHg, with either criterion sufficient for eligibility. This study-specific definition encompassed multiple blood pressure categories under the 2018 Korean Society of Hypertension (KSH) guidelines [24]. However, individuals who were receiving antihypertensive medication or required pharmacological treatment for hypertension were excluded from the study.

The main exclusion criteria included regular consumption of ginseng or red ginseng products or a history of hypersensitivity to ginseng; use of antihypertensive medications or other medications or supplements that could affect blood pressure or circulation; recent use of herbal medicines, corticosteroids, or hormonal agents; diabetes requiring pharmacological treatment, thyroid dysfunction, or other clinically significant hepatic, renal, or medical conditions that could affect study participation or outcome assessment; recent surgery or a history of gastrointestinal surgery that could affect absorption; pregnancy or breastfeeding; recent participation in another clinical trial or blood donation; and uncontrolled hypertension or recent initiation of antihypertensive medication.

### 2.3. Study Design and Randomization

This 12-week, randomized, double-blind, placebo-controlled clinical trial was conducted at Dankook University Hospital, Republic of Korea, between 9 November 2020 and 7 October 2021. Eligible participants were randomly assigned to one of three groups: the vitamin C-treated white ginseng (VTWG), enzyme-treated red ginseng (ETRG), or placebo group.

Randomization numbers for 90 participants were generated by the study sponsor using an automated randomization program (Randomization.com). The clinical research coordinator was responsible for participant enrollment and intervention assignment, with randomization numbers assigned sequentially according to the order of enrollment. Allocation concealment was maintained using sealed, opaque envelopes, and investigators and recruitment personnel did not have access to the allocation sequence.

To maintain double blinding, the study products were prepared to be indistinguishable in size, shape, color, odor, texture, labeling, and packaging. Participants received the study products according to their assigned randomization numbers, and access to the randomization code was restricted until completion of the study.

Participants completed four study visits: screening at week −4 (Visit 1), enrollment and baseline assessment at week 0 (Visit 2), an interim assessment at week 6 ± 7 days (Visit 3), and the final efficacy and safety assessment at week 12 ± 7 days (Visit 4).

### 2.4. Study Products and Interventions

The study products were supplied by BTGin Co., Ltd. (Daejeon, Republic of Korea). Two differently processed Rg3-enriched Korean ginseng preparations were used: VTWG and ETRG.

VTWG was prepared by drying fresh Korean ginseng, followed by treatment with a 1.0% vitamin C solution and extraction with 50% ethanol, as previously described [16]. The extract was concentrated under vacuum and prepared as a powder. ETRG was prepared from Korean red ginseng (*Panax ginseng* C.A. Meyer) by ethanol extraction, followed by enzyme-assisted acid hydrolysis to enhance the conversion of ginsenosides to Rg3, as previously described [14,15]. The resulting extract was purified using DIAION HP20 resin and concentrated under vacuum. The Rg3 contents of VTWG and ETRG were 15 and approximately 100 mg/g, respectively, as determined by high-performance liquid chromatography (HPLC).

All study products were provided as 425 mg tablets. The VTWG tablets contained 52.9% VTWG, 46.9% crystalline cellulose, and 0.2% ginseng flavor; the ETRG tablets contained 7.8% ETRG, 92.0% crystalline cellulose, and 0.2% ginseng flavor; and the placebo tablets contained 99.8% crystalline cellulose and 0.2% ginseng flavor. Participants were instructed to consume six tablets twice daily, in the morning and evening after meals, for 12 weeks. This corresponded to daily intakes of approximately 2700 mg of VTWG and 400 mg of ETRG. Despite the different Rg3 concentrations of the two preparations, both interventions provided approximately 40 mg/day of Rg3 (Table 1). Participants were instructed to maintain their usual dietary intake and lifestyle habits throughout the 12-week intervention period.

### 2.5. Efficacy Outcome Measures

The primary efficacy outcomes were changes in SBP and DBP. The secondary efficacy outcomes included mean arterial pressure (MAP), pulse rate, high-sensitivity C-reactive protein (hs-CRP), and NO.

Blood pressure was measured using a BPBIO320 blood pressure monitor (Inbody Co., Ltd., Seoul, Republic of Korea) with participants in a seated position after at least 5 min of rest [25]. An appropriate cuff size was used for each participant. All blood pressure measurements were performed by study personnel blinded to group allocation. Two measurements were obtained at 2 min intervals. If the two readings differed by 10 mmHg or more, an additional measurement was obtained, and the mean of the final two readings was used for analysis. Blood pressure measurements obtained at screening (Visit 1, week −4) were used to determine eligibility and served as the baseline values for efficacy analyses. MAP was calculated from SBP and DBP using the formula MAP = DBP + (SBP − DBP)/3. The calculated MAP values were verified by recalculation before statistical analysis. Changes in all primary and secondary efficacy outcomes from baseline to week 12 were evaluated and compared between each intervention group and the placebo group.

### 2.6. Safety Outcome Measures

Safety assessments included the evaluation of adverse events (AEs) and clinical laboratory parameters. AEs were monitored throughout the study based on participant reports and were documented during the study period.

Clinical laboratory assessments included hematological, biochemical, and urinalysis parameters. Blood samples for laboratory assessments were collected after an overnight fast of at least 12 h. Hematological parameters included white blood cell (WBC) count, red blood cell (RBC) count, hemoglobin, hematocrit, platelet count, neutrophils, lymphocytes, monocytes, eosinophils, and basophils. Biochemical parameters included total bilirubin, alkaline phosphatase (ALP), γ-glutamyl transferase (γ-GT), alanine aminotransferase (ALT), aspartate aminotransferase (AST), total cholesterol, triglycerides, high-density lipoprotein cholesterol (HDL-C), low-density lipoprotein cholesterol (LDL-C), glucose, total protein, albumin, blood urea nitrogen (BUN), creatinine, creatine kinase (CK), and lactate dehydrogenase (LDH). Urinalysis was also performed.

### 2.7. Statistical Analysis

The sample size was determined to evaluate the effects of the two Rg3-enriched Korean ginseng preparations on blood pressure compared with placebo. The required sample size was estimated based on a previous clinical study with a similar study design and primary efficacy outcomes [26]. With a two-sided significance level of 5% and a statistical power of 80%, the estimated sample size was 24 participants per group. To account for a potential dropout rate of 20%, 30 participants per group were scheduled for randomization, resulting in a total sample size of 90 participants.

All statistical analyses were performed using R software (version 4.0.3) and SAS software (version 9.4; SAS Institute Inc., Cary, NC, USA). All statistical tests were two-sided, and statistical significance was set at *p* < 0.05. Continuous variables are presented as mean ± standard deviation (SD), and categorical variables are presented as number (n) and percentage (%). Baseline characteristics among the three groups were compared using analysis of variance (ANOVA) or the Kruskal–Wallis test for continuous variables and the chi-square test for categorical variables, as appropriate.

Statistical analyses were conducted according to the prespecified study protocol. The per-protocol set (PPS) was prespecified as the primary analysis population for efficacy and included participants who completed the study in accordance with the protocol, maintained an overall compliance of ≥70%, and had no major protocol deviations affecting efficacy evaluation. The PPS was selected to evaluate the efficacy of the interventions under conditions of adequate adherence to the study protocol. Additional efficacy analyses were conducted using the full analysis set (FAS) to assess the consistency of the findings.

Within-group changes from baseline to week 12 were analyzed using paired *t*-tests. Between-group differences in changes from baseline were analyzed using independent *t*-tests for each intervention group versus the placebo group. Additionally, analysis of covariance (ANCOVA) was performed to estimate adjusted mean differences in changes from baseline, 95% confidence intervals (CIs), and *p*-values between each intervention group and the placebo group, adjusting for the baseline value of each outcome.

## 3. Results

### 3.1. Study Population and Baseline Characteristics

**Figure 1:**
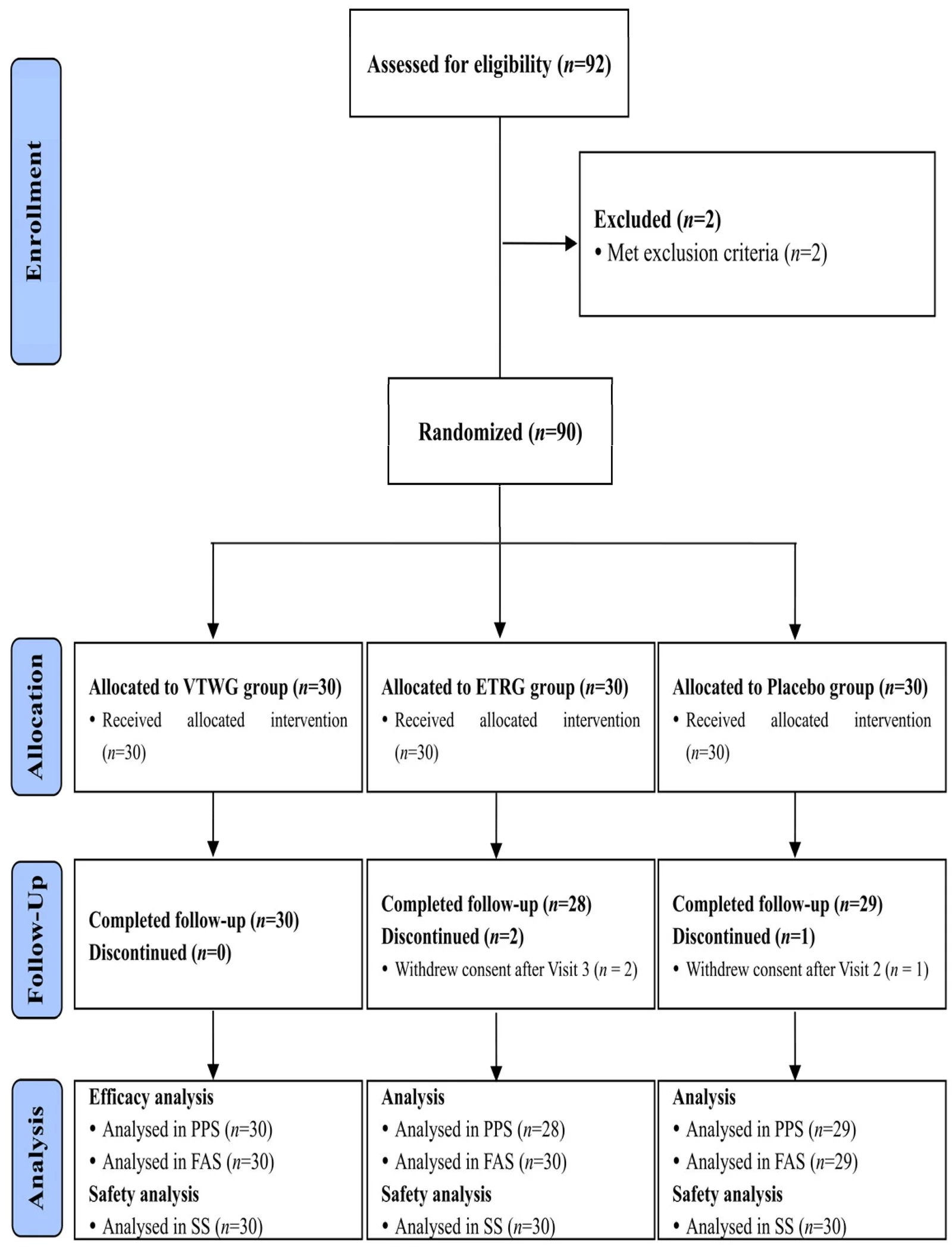
CONSORT flow diagram of participant enrollment, allocation, follow-up, and analysis.

A total of 90 participants with early-stage hypertension were randomly assigned to three groups (*n* = 30 per group). During the study, three participants withdrew their consent and discontinued the study. Two participants in the ETRG group withdrew after Visit 3 (week 6), whereas one participant in the placebo group withdrew after Visit 2 (week 0), approximately 7 weeks after randomization. Consequently, 87 participants (VTWG, *n* = 30; ETRG, *n* = 28; placebo, *n* = 29) were included in the PPS for the efficacy analyses. The FAS included 89 participants (VTWG, *n* = 30; ETRG, *n* = 30; placebo, *n* = 29), excluding one participant in the placebo group who had no post-baseline efficacy data. The flow of participants throughout the study is shown in Figure 1.

Overall compliance with study product administration was high, with mean compliance rates of 96.33 ± 4.75%, 95.59 ± 5.48%, and 96.02 ± 5.88% in the VTWG, ETRG, and placebo groups, respectively. No significant difference was observed among the three groups (*p* = 0.871). No participants were excluded from the PPS due to insufficient compliance.

The baseline characteristics of the three groups are presented in Table 2. According to the 2018 KSH guidelines, 17 participants in the VTWG group, 8 in the ETRG group, and 5 in the placebo group were classified as having grade 1 hypertension at baseline. Significant differences among the three groups were observed in age, SBP, and pulse rate at baseline (*p* = 0.001, *p* < 0.001, and *p* = 0.002, respectively), whereas no significant differences were observed in the other baseline characteristics.

### 3.2. Primary Efficacy Outcomes

#### 3.2.1. Systolic Blood Pressure

**Figure 2:**
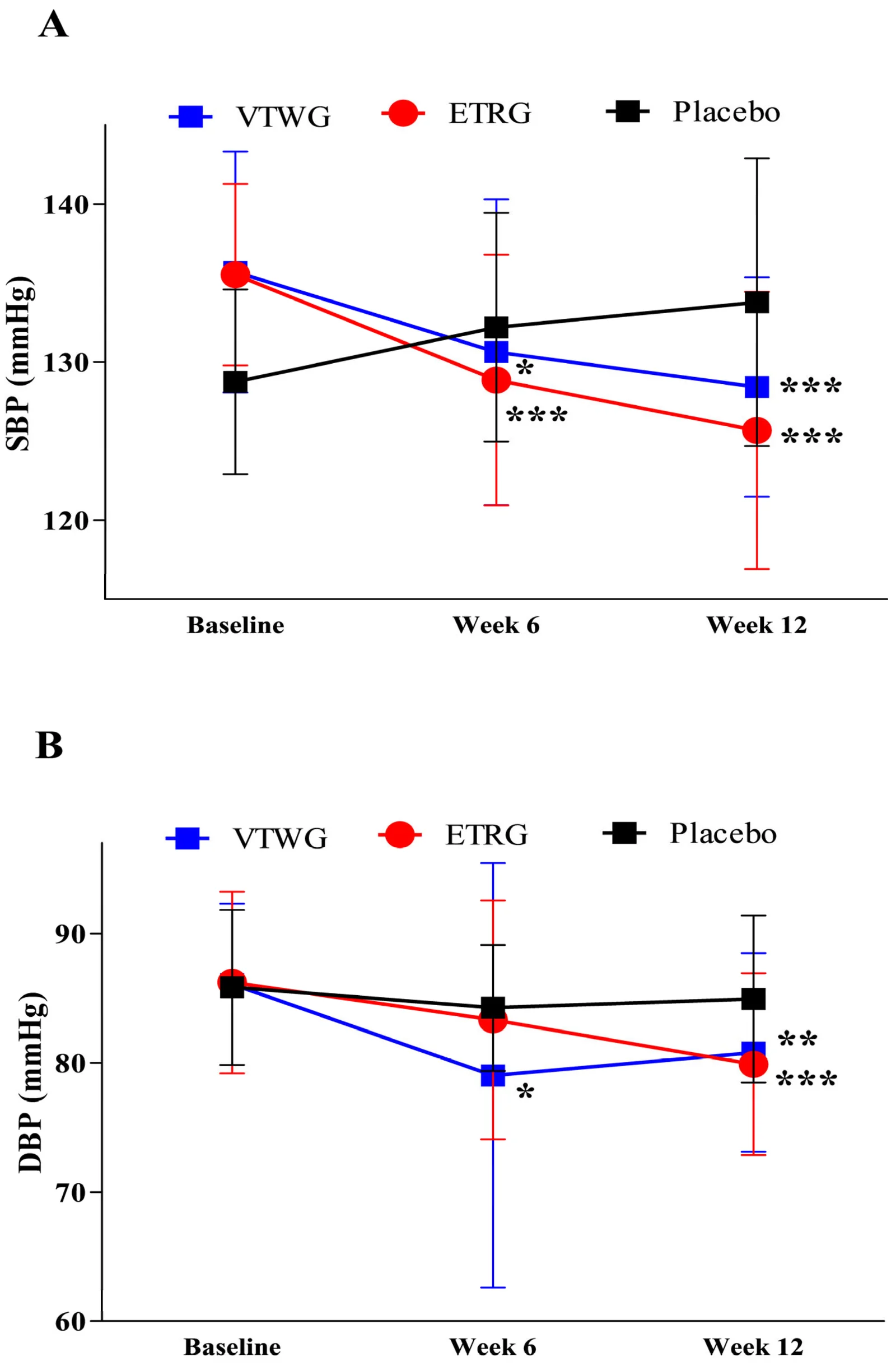
Changes in blood pressure parameters over the 12-week intervention period: (**A**) Systolic blood pressure (SBP) and (**B**) diastolic blood pressure (DBP). Values are presented as mean ± SD at baseline, week 6, and week 12. Between-group differences in changes from baseline at weeks 6 and 12 were analyzed using ANCOVA adjusted for the baseline value of each outcome. * *p* < 0.05, ** *p* < 0.01, *** *p* < 0.001 vs. placebo group.

Changes in SBP from baseline to week 12 are presented in Table 3, and SBP values at each study visit are illustrated in Figure 2A. In the VTWG group, SBP decreased significantly from 135.70 ± 7.62 mmHg at baseline to 128.43 ± 6.94 mmHg at week 12, corresponding to a change of −7.27 ± 6.15 mmHg (*p* < 0.0001). In the ETRG group, SBP also decreased significantly from 135.54 ± 5.74 mmHg to 125.68 ± 8.77 mmHg, corresponding to a change of −9.86 ± 6.47 mmHg (*p* < 0.0001). In contrast, SBP in the placebo group increased from 128.76 ± 5.84 mmHg to 133.79 ± 9.10 mmHg, with a change of 5.03 ± 6.52 mmHg (*p* = 0.0003). The changes in SBP were significantly different between the VTWG and placebo groups (*p* < 0.0001) and between the ETRG and placebo groups (*p* < 0.0001). Additional ANCOVA adjusting for baseline SBP showed significant between-group differences in SBP changes for both VTWG (adjusted mean difference: 10.72 mmHg; 95% CI: 7.08–14.36; *p* < 0.0001) and ETRG (15.32 mmHg; 95% CI: 11.27–19.36; *p* < 0.0001) compared with placebo.

#### 3.2.2. Diastolic Blood Pressure

Changes in DBP from baseline to week 12 are presented in Table 3, and DBP values at each study visit are illustrated in Figure 2B. In the VTWG group, DBP decreased significantly from 86.07 ± 6.22 mmHg at baseline to 80.80 ± 7.67 mmHg at week 12, corresponding to a change of −5.27 ± 5.58 mmHg (*p* < 0.0001). In the ETRG group, DBP also decreased significantly from 86.21 ± 7.03 mmHg to 79.89 ± 7.03 mmHg, corresponding to a change of −6.32 ± 5.55 mmHg (*p* < 0.0001). In the placebo group, DBP changed from 85.83 ± 5.98 mmHg to 84.93 ± 6.45 mmHg, corresponding to a change of −0.90 ± 6.31 mmHg (*p* = 0.451). The changes in DBP were significantly different between the VTWG and placebo groups (*p* = 0.007) and between the ETRG and placebo groups (*p* = 0.001). Additional ANCOVA adjusting for baseline DBP showed significant between-group differences in DBP changes for both VTWG (adjusted mean difference: 4.30 mmHg; 95% CI: 1.32–7.28; *p* = 0.006) and ETRG (5.28 mmHg; 95% CI: 2.38–8.18; *p* = 0.0006) compared with placebo.

### 3.3. Secondary Efficacy Outcomes

#### 3.3.1. Mean Arterial Pressure

Changes in MAP from baseline to week 12 are presented in Table 4. In the VTWG group, MAP decreased significantly from 102.37 ± 5.38 mmHg at baseline to 96.57 ± 6.55 mmHg at week 12, corresponding to a change of −5.80 ± 4.44 mmHg (*p* < 0.0001). In the ETRG group, MAP also decreased significantly from 102.43 ± 5.61 mmHg to 94.89 ± 7.04 mmHg, corresponding to a change of −7.54 ± 5.28 mmHg (*p* < 0.0001). In the placebo group, MAP changed from 100.00 ± 4.94 mmHg to 101.38 ± 6.19 mmHg, corresponding to a change of 1.38 ± 5.19 mmHg (*p* = 0.163). The changes in MAP were significantly different between the VTWG and placebo groups (*p* < 0.0001) and between the ETRG and placebo groups (*p* < 0.0001). Additional ANCOVA adjusting for baseline MAP also showed significant differences for VTWG (adjusted mean difference: 6.77 mmHg; 95% CI: 4.21–9.33; *p* < 0.0001) and ETRG (8.42 mmHg; 95% CI: 5.60–11.24; *p* < 0.0001) compared with placebo.

#### 3.3.2. Pulse Rate

Changes in pulse rate from baseline to week 12 are presented in Table 4. Pulse rate decreased from baseline to week 12 in all groups; however, the changes did not differ significantly between the VTWG and placebo groups (*p* = 0.669) or between the ETRG and placebo groups (*p* = 0.479). Additional ANCOVA adjusting for baseline pulse rate showed no significant differences between VTWG and placebo (adjusted mean difference: 1.48 beats/min; 95% CI: −3.87 to 6.84; *p* = 0.582) or between ETRG and placebo (3.47 beats/min; 95% CI: −1.81 to 8.74; *p* = 0.193).

#### 3.3.3. High-Sensitivity C-Reactive Protein

Changes in hs-CRP from baseline to week 12 are presented in Table 4. No significant changes in hs-CRP were observed from baseline to week 12 in any group. The changes did not differ significantly between the VTWG and placebo groups (*p* = 0.760) or between the ETRG and placebo groups (*p* = 0.596). Additional ANCOVA adjusting for baseline hs-CRP showed no significant differences between VTWG and placebo (adjusted mean difference: −0.13 mg/L; 95% CI: −0.54 to 0.28; *p* = 0.537) or between ETRG and placebo (−0.10 mg/L; 95% CI: −0.49 to 0.29; *p* = 0.619).

#### 3.3.4. Nitric Oxide

Changes in NO levels from baseline to week 12 are presented in Table 4. NO levels increased slightly from baseline to week 12 in all groups; however, the within-group changes were not statistically significant. The changes did not differ significantly between the VTWG and placebo groups (*p* = 0.836) or between the ETRG and placebo groups (*p* = 0.507). However, additional ANCOVA adjusting for baseline NO showed a significant difference between ETRG and placebo (adjusted mean difference: −16.22 μmol/L; 95% CI: −29.36 to −3.08; *p* = 0.017), but not between VTWG and placebo (−12.78 μmol/L; 95% CI: −29.50 to 3.93; *p* = 0.131).

Additional analyses using the FAS yielded results consistent with those of the PPS analysis (Supplementary Tables S1 and S2). Significant between-group differences in SBP, DBP, and MAP changes were maintained for both intervention groups compared with placebo (all adjusted *p* < 0.01). For NO, a significant between-group difference was observed only between ETRG and placebo (adjusted *p* = 0.014).

### 3.4. Safety Outcomes

Safety was evaluated based on AEs and clinical laboratory parameters. A total of 18 AEs were reported during the study: five in the VTWG group, seven in the ETRG group, and six in the placebo group. Of these, three were classified as serious adverse events (SAEs): left chronic otitis media, colon polyp, and rupture of the anterior cruciate ligament. None of the reported AEs, including the three SAEs, were considered related to the study products.

Clinical laboratory parameters, including hematological, biochemical, and urinalysis measures, showed no clinically meaningful changes during the study. Overall, 12-week supplementation with VTWG or ETRG was well tolerated, with no clinically relevant safety concerns identified.

## 4. Discussion

In this randomized, double-blind, placebo-controlled trial, 12-week supplementation with either VTWG or ETRG significantly reduced SBP and DBP compared with placebo in adults with early-stage hypertension. MAP, a secondary efficacy outcome, was also significantly reduced in both intervention groups compared with placebo. Notably, consistent blood pressure-lowering effects were observed with both Rg3-enriched preparations despite their different processing methods. These findings extend the currently limited clinical evidence on Rg3-enriched ginseng by demonstrating significant blood pressure reductions following 12 weeks of supplementation with two differently processed Rg3-enriched Korean ginseng preparations in adults with early-stage hypertension.

Previous clinical studies of conventional ginseng or KRG have reported inconsistent effects on blood pressure and vascular function [17,18,19,20]. A meta-analysis of randomized controlled trials found no significant overall effects of ginseng on SBP, DBP, or MAP [27], whereas a subsequent systematic review and meta-analysis focusing on individuals with prehypertension or hypertension reported modest reductions in SBP and DBP with long-term KRG supplementation [20]. In a 12-week randomized controlled trial in prehypertensive adults, Cha et al. reported reductions in SBP and DBP following conventional KRG supplementation [26]. These findings suggest that blood pressure responses to ginseng may vary according to the study population as well as the composition and processing of the ginseng preparation. Notably, the magnitude of the SBP reduction relative to placebo observed in the present study was greater than that generally reported in previous ginseng studies and meta-analyses [20,27]. Several factors may have contributed to this difference, including the use of Rg3-enriched preparations, differences in ginsenoside composition and dosage, and variations in study populations and baseline blood pressure characteristics. Therefore, the relatively large treatment effects should be interpreted cautiously and confirmed in larger, independent clinical trials. Importantly, clinical evidence specifically evaluating Rg3-enriched ginseng preparations remains limited. In a randomized, double-blind, crossover trial in healthy young adults, a single dose of Rg3-enriched KRG reduced central and brachial SBP and DBP, as well as MAP, compared with control [21]. However, this study assessed only the acute effects of a single administration. Subsequently, 12-week co-administration of Rg3-enriched KRG and American ginseng improved blood pressure-related outcomes in individuals with type 2 diabetes and hypertension receiving standard medical therapy [22,23]. Because Rg3-enriched KRG was administered in combination with American ginseng in that study, its independent long-term effects could not be determined. The present study therefore extends previous clinical evidence by demonstrating significant reductions in SBP, DBP, and MAP after 12 weeks of supplementation with Rg3-enriched Korean ginseng preparations administered without another ginseng preparation in adults with early-stage hypertension.

An important feature of the present study was the evaluation of two Rg3-enriched Korean ginseng preparations produced using different processing methods. VTWG and ETRG differed substantially in their Rg3 concentrations (15 and approximately 100 mg/g, respectively), but the daily amounts of the preparations were adjusted to provide a comparable Rg3 intake of approximately 40 mg/day. Despite differences in processing methods and ginseng matrices, both preparations significantly reduced SBP, DBP, and MAP compared with placebo. The observation of blood pressure reductions with both preparations is noteworthy, as previous clinical studies and meta-analyses have suggested that blood pressure responses may vary across different ginseng types and preparations [17,20,27]. This consistent pattern of blood pressure reduction across the two preparations supports the potential relevance of Rg3 enrichment in the development of ginseng preparations for blood pressure management. However, because the study was not designed to compare VTWG directly with ETRG and did not include a conventional ginseng or Rg3-free ginseng comparator, the observed effects cannot be attributed specifically to Rg3 or interpreted as evidence of equivalent efficacy between the two preparations.

The blood pressure-lowering effects observed in the present study are biologically plausible based on the known vascular actions of Rg3. Experimental studies have shown that Rg3 promotes vasorelaxation through mechanisms involving activation of the PI3K/Akt/eNOS pathway and increased NO production [10,12]. Consistent with these mechanisms, preclinical studies of Rg3-enriched ginseng preparations have demonstrated enhanced eNOS phosphorylation and NO production, improved vasorelaxation, and reductions in blood pressure in hypertensive animal models [14,15,16]. Human studies also provide evidence that ginseng may influence endothelial function. In a randomized controlled trial in healthy individuals, KRG improved flow-mediated dilation, suggesting an acute effect on endothelial function [28]. More recently, a meta-analysis of 13 randomized controlled trials reported improvements in flow-mediated dilation and pulse wave velocity following ginseng supplementation [29]. In the present study, circulating NO levels did not differ significantly between either intervention group and the placebo group in the original analysis. However, an additional ANCOVA adjusting for baseline NO levels revealed a significant between-group difference in NO changes between the ETRG and placebo groups (adjusted mean difference: −16.22 μmol/L; 95% CI: −29.36 to −3.08; *p* = 0.017), whereas no significant difference was observed for VTWG. Although this finding is consistent with the potential involvement of NO in the vascular effects of Rg3, it should be interpreted cautiously because statistical significance was observed only in the additional analysis. Evidence regarding the effects of ginseng on circulating NO in humans remains limited. Although the recent meta-analysis reported a significant pooled increase in endothelium-derived NO, this analysis was based on only four effect sizes from three studies, and the statistical significance was not robust in sensitivity analyses [29]. Taken together, these findings suggest a possible involvement of NO-related pathways in the observed blood pressure reductions. However, the inconsistency between the original and additional analyses, together with the limited evidence from human studies, precludes definitive conclusions regarding an NO-mediated mechanism.

The findings of the present study may be particularly relevant to adults with early-stage hypertension, in whom lifestyle modification remains an important component of blood pressure management. The reductions in SBP, DBP, and MAP observed with both preparations are noteworthy because even modest reductions in blood pressure have been associated with lower cardiovascular risk at the population level [30]. In addition, both Rg3-enriched ginseng preparations were well tolerated, with no treatment-related AEs or clinically relevant laboratory abnormalities during the 12-week intervention. These safety findings are also consistent with previous clinical studies in which KRG or Rg3-enriched ginseng preparations were generally well tolerated [21,22,23,26]. Taken together, the blood pressure and safety findings support further investigation of Rg3-enriched Korean ginseng preparations for blood pressure management in adults with early-stage hypertension.

This study has several strengths, including its randomized, double-blind, placebo-controlled design; the evaluation of two differently processed Rg3-enriched Korean ginseng preparations providing a comparable daily amount of Rg3; and the assessment of multiple blood pressure outcomes. Nevertheless, several limitations should be considered. First, the study was conducted at a single center with a relatively modest sample size and a 12-week intervention period, which may limit the generalizability of the findings and preclude conclusions regarding longer-term efficacy and safety. In addition, the study-specific eligibility criteria encompassed multiple blood pressure categories under the 2018 KSH guidelines, which should be considered when interpreting the findings and their generalizability. Second, some baseline characteristics differed among the groups. Although ANCOVA adjusting for the baseline value of each outcome showed significant between-group differences in SBP, DBP, and MAP changes, potential residual confounding due to other baseline imbalances cannot be excluded. In addition, the use of screening blood pressure measurements as baseline values, together with the observed increase in SBP in the placebo group, suggests that regression to the mean may have influenced the observed blood pressure changes. Therefore, its potential contribution to the treatment effects cannot be excluded. Third, the absence of a conventional ginseng or Rg3-free comparator and a dose-ranging design precludes determination of the independent contribution of Rg3 and assessment of dose–response relationships. Finally, the inconsistent findings regarding circulating NO across statistical models preclude definitive conclusions regarding the mechanisms underlying the observed blood pressure effects. In addition, the trial was registered retrospectively. Although IRB approval was obtained before participant enrollment, the delayed registration represents a methodological limitation in terms of research transparency.

Despite these limitations, the consistent reductions in SBP, DBP, and MAP observed with two differently processed Rg3-enriched preparations provide clinically relevant evidence that supports further investigation of these preparations for blood pressure management in adults with early-stage hypertension.

## 5. Conclusions

This randomized, double-blind, placebo-controlled clinical trial showed that 12-week supplementation with either VTWG or ETRG significantly reduced SBP, DBP, and MAP in adults with early-stage hypertension compared with placebo. Although the two preparations differed in their processing methods and Rg3 concentrations, both provided a comparable daily amount of Rg3 (approximately 40 mg/day) and showed consistent blood pressure-lowering effects. Both preparations were well tolerated, with no treatment-related AEs or clinically relevant laboratory abnormalities during the 12-week intervention. Overall, these findings provide clinical evidence that Rg3-enriched Korean ginseng preparations may have potential for blood pressure management in adults with early-stage hypertension.

## Figures and Tables

**Table 1:** Composition and daily intake of the study products.

Group	Ingredients	Composition (%)	Daily Intake (mg/Day)	Rg3 Intake (mg/Day)
VTWG	VTWG Crystalline cellulose Ginseng flavor	52.9 46.9 0.2	2698 2392 10	40 — —
	Total	100	5100	40
ETRG	ETRG Crystalline cellulose Ginseng flavor	7.8 92.0 0.2	398 4692 10	40 — —
	Total	100	5100	40
Placebo	Crystalline cellulose Ginseng flavor	99.8 0.2	5090 10	— —
	Total	100	5100	—

Participants consumed 12 tablets (425 mg/tablet) daily, corresponding to a total daily intake of 5100 mg.

**Table 2:** Baseline demographic characteristics of the randomized participants.

Variable	VTWG Group (*n* = 30)	ETRG Group (*n* = 30)	Placebo Group (*n* = 30)	*p*-Value ^(1)^
Sex (M/F)	23/7	22/8	20/10	0.679 ^(2)^
Age (years)	39.3 ± 11.7	42.9 ± 9.6	32.6 ± 11.0	0.001
Height (cm)	170.3 ± 7.9	170.6 ± 9.0	171.8 ± 8.8	0.878
Weight (kg)	76.1 ± 11.7	73.5 ± 9.3	73.5 ± 15.1	0.650
BMI (kg/m^2^)	26.2 ± 3.6	25.3 ± 2.5	24.7 ± 3.3	0.164
SBP (mmHg)	135.7 ± 7.6	135.2 ± 5.8	128.9 ± 5.8	<0.001
DBP (mmHg)	86.1 ± 6.2	85.9 ± 7.0	85.8 ± 5.9	0.862
Pulse Rate (beats/min)	82.6 ± 10.8	76.6 ± 11.4	86.9 ± 11.2	0.002
Drinking (Yes/No)	23/7	28/2	26/4	0.123 ^(2)^
Smoking (Yes/No)	10/20	5/25	9/21	0.303 ^(2)^
Exercise (Yes/No)	25/5	24/6	20/10	0.271 ^(2)^

Values are presented as mean ± SD or n; ^(1)^ Continuous variables were compared among the three groups using one-way ANOVA or the Kruskal–Wallis test, as appropriate; ^(2)^ Categorical variables were compared using the chi-square test. Abbreviations: BMI, body mass index; SBP, systolic blood pressure; DBP, diastolic blood pressure.

**Table 3:** Changes in primary efficacy outcomes from baseline to week 12 (PPS analysis).

Variable	Group	Baseline	Week 12	Change from Baseline	*p*-Value ^(1)^ (Within-Group)	*p*-Value ^(2)^ (vs. Placebo)	Adjusted Mean Difference [95% CI] ^(3)^	Adjusted *p*-Value ^(3)^ (vs. Placebo)
SBP (mmHg)	VTWG group (*n* = 30)	135.70 ± 7.62	128.43 ± 6.94	−7.27 ± 6.15	<0.0001	<0.0001	10.72 [7.08, 14.36]	<0.0001
	ETRG group (*n* = 28)	135.54 ± 5.74	125.68 ± 8.77	−9.86 ± 6.47	<0.0001	<0.0001	15.32 [11.27, 19.36]	<0.0001
	Placebo group (*n* = 29)	128.76 ± 5.84	133.79 ± 9.10	5.03 ± 6.52	0.0003	—	—	—
DBP (mmHg)	VTWG group (*n* = 30)	86.07 ± 6.22	80.80 ± 7.67	−5.27 ± 5.58	<0.0001	0.007	4.30 [1.32, 7.28]	0.006
	ETRG group (*n* = 28)	86.21 ± 7.03	79.89 ± 7.03	−6.32 ± 5.55	<0.0001	0.001	5.28 [2.38, 8.18]	0.0006
	Placebo group (*n* = 29)	85.83 ± 5.98	84.93 ± 6.45	−0.90 ± 6.31	0.451	—	—	—

Values are presented as mean ± SD. ^(1)^ Within-group differences between baseline and week 12 were analyzed using a paired *t*-test. ^(2)^ Between-group differences in changes from baseline were analyzed using an independent *t*-test for VTWG vs. placebo and ETRG vs. placebo, respectively. ^(3)^ Adjusted mean differences (95% CI) and *p*-values were obtained using ANCOVA adjusted for the baseline value of each outcome, with differences calculated as placebo minus intervention. Abbreviations: SBP, systolic blood pressure; DBP, diastolic blood pressure; ANCOVA, analysis of covariance; CI, confidence interval.

**Table 4:** Changes in secondary efficacy outcomes from baseline to week 12 (PPS analysis).

Variable	Group	Baseline	Week 12	Change from Baseline	*p*-Value ^(1)^ (Within-Group)	*p*-Value ^(2)^ (vs. Placebo)	Adjusted Mean Difference [95% CI] ^(3)^	Adjusted *p*-Value ^(3)^ (vs. Placebo)
MAP (mmHg)	VTWG group (*n* = 30)	102.37 ± 5.38	96.57 ± 6.55	−5.80 ± 4.44	<0.0001	<0.0001	6.77 [4.21, 9.33]	<0.0001
	ETRG group (*n* = 28)	102.43 ± 5.61	94.89 ± 7.04	−7.54 ± 5.28	<0.0001	<0.0001	8.42 [5.60, 11.24]	<0.0001
	Placebo group (*n* = 29)	100.00 ± 4.94	101.38 ± 6.19	1.38 ± 5.19	0.163	—	—	—
Pulse rate (beats/min)	VTWG group (*n* = 30)	82.60 ± 10.80	78.30 ± 10.16	−4.30 ± 11.60	0.052	0.669	1.48 [−3.87, 6.84]	0.582
	ETRG group (*n* = 28)	76.54 ± 11.75	72.89 ± 9.50	−3.64 ± 8.27	0.028	0.479	3.47 [−1.81, 8.74]	0.193
	Placebo group (*n* = 29)	87.14 ± 11.30	81.48 ± 11.40	−5.66 ± 12.64	0.023	—	—	—
hs-CRP (mg/L)	VTWG group (*n* = 30)	1.06 ± 1.01	1.05 ± 0.97	−0.01 ± 0.85	0.956	0.760	−0.13 [−0.54, 0.28]	0.537
	ETRG group (*n* = 28)	0.91 ± 0.73	0.95 ± 0.75	0.04 ± 0.73	0.766	0.596	−0.10 [−0.49, 0.29]	0.619
	Placebo group (*n* = 29)	0.96 ± 1.00	0.87 ± 0.87	−0.08 ± 1.01	0.660	—	—	—
Nitric oxide (μmol/L)	VTWG group (*n* = 30)	50.71 ± 34.09	54.29 ± 38.99	3.58 ± 45.76	0.671	0.836	−12.78 [−29.50, 3.93]	0.131
	ETRG group (*n* = 28)	49.61 ± 19.69	56.02 ± 25.29	6.41 ± 30.54	0.276	0.507	−16.22 [−29.36, −3.08]	0.017
	Placebo group (*n* = 29)	36.48 ± 13.25	38.10 ± 20.25	1.62 ± 23.12	0.708	—	—	—

Values are presented as mean ± SD. ^(1)^ Within-group differences between baseline and week 12 were analyzed using a paired *t*-test. ^(2)^ Between-group differences in changes from baseline were analyzed using an independent *t*-test for VTWG vs. placebo and ETRG vs. placebo, respectively. ^(3)^ Adjusted mean differences (95% CI) and *p*-values were obtained using ANCOVA adjusted for the baseline value of each outcome, with differences calculated as placebo minus intervention. Abbreviations: MAP, mean arterial pressure; hs-CRP, high-sensitivity C-reactive protein; NO, nitric oxide; ANCOVA, analysis of covariance; CI, confidence interval.

## Data Availability

The datasets generated and analyzed during the current study are available from the corresponding author upon reasonable request. The data are not publicly available due to privacy and ethical restrictions.

## Supplementary Materials

The following supporting information can be downloaded at https://www.mdpi.com/article/10.3390/nu18203448/s1: Table S1. Changes in primary efficacy outcomes from baseline to week 12 (FAS analysis). Table S2. Changes in secondary efficacy outcomes from baseline to week 12 (FAS analysis).

## Abbreviations

The following abbreviations are used in this manuscript:

VTWG	vitamin C-treated white ginseng
ETRG	enzyme-treated red ginseng
SBP	systolic blood pressure
DBP	diastolic blood pressure
MAP	mean arterial pressure
CVD	cardiovascular disease
NO	nitric oxide
eNOS	endothelial nitric oxide synthase
KRG	Korean red ginseng
IRB	Institutional Review Board
CRIS	Clinical Research Information Service
KSH	Korean Society of Hypertension
HPLC	high-performance liquid chromatography
hs-CRP	high-sensitivity C-reactive protein
AEs	adverse events
WBC	white blood cell
RBC	red blood cell
ALP	alkaline phosphatase
γ-GT	γ-glutamyl transferase
ALT	alanine aminotransferase
AST	aspartate aminotransferase
HDL-C	high-density lipoprotein cholesterol
LDL-C	low-density lipoprotein cholesterol
BUN	blood urea nitrogen
CK	creatine kinase
LDH	lactate dehydrogenase
SD	standard deviation
ANOVA	analysis of variance
PPS	per-protocol set
FAS	full analysis set
ANCOVA	analysis of covariance
CI	confidence interval
BMI	body mass index
