# Supplementary material for: Effects of Two Rg3-Enriched Korean Ginseng Preparations on Blood Pressure in Adults with Early-Stage Hypertension: A Randomized, Double-Blind, Placebo-Controlled Trial
Source: Nutrients. 2026 Oct 20;18(20):3448. doi: 10.3390/nu18203448 (PMC13708105; doi:10.3390/nu18203448)
Supplement: Supplementary file 1 [file nutrients-18-03448-s001.zip › nutrients-4596985-supplementary.pdf]

Table S1. Changes in primary efficacy outcomes from baseline to week 12 (FAS analysis).

Variable	Group	Baseline	Week 12	Change from baseline	*p*-value [(1)] (within-group)	*p*-value [(2)] (vs. placebo)	Adjusted mean difference [95% CI] [(3)]	Adjusted *p*-value [(3)] (vs. placebo)
SBP (mmHg)	VTWG group (*n*=30)	135.70 ± 7.62	128.43 ± 6.94	-7.27 ± 6.15	<0.0001	<0.0001	10.72 [7.08, 14.36]	<0.0001
	ETRG group (*n*=30)	135.20 ± 5.76	126.00 ± 8.60	-9.20 ± 6.73	<0.0001	<0.0001	14.31 [10.31, 18.32]	<0.0001
	Placebo group (*n*=29)	128.76 ± 5.84	133.79 ± 9.10	5.03 ± 6.52	0.0003	—	—	—
DBP (mmHg)	VTWG group (*n*=30)	86.07 ± 6.22	80.80 ± 7.67	-5.27 ± 5.58	<0.0001	0.007	4.30 [1.32, 7.28]	0.006
	ETRG group (*n*=30)	85.87 ± 6.95	79.97 ± 6.83	-5.90 ± 5.59	<0.0001	0.002	4.99 [2.16, 7.81]	0.0008
	Placebo group (*n*=29)	85.83 ± 5.98	84.93 ± 6.45	-0.90 ± 6.31	0.451	—	—	—

Values are presented as mean ± SD; [(1)] Within-group differences between baseline and week 12 were analyzed using a paired t-test; [(2)] Between-group differences in changes from baseline were analyzed using an independent t-test for VTWG vs. placebo and ETRG vs. placebo, respectively; [(3)] Adjusted mean differences (95% CI) and p-values were obtained using ANCOVA adjusted for the baseline value of each outcome, with differences calculated as placebo minus intervention. Abbreviations: SBP, systolic blood pressure; DBP, diastolic blood pressure; ANCOVA, analysis of covariance; CI, confidence interval.

Table S2. Changes in secondary efficacy outcomes from baseline to week 12 (FAS analysis).

Variable	Group	Baseline	Week 12	Change from baseline	*p*-value (1) (within-group)	*p*-value (2) (vs. placebo)	Adjusted mean difference [95% CI] (3)	Adjusted *p*-value (3) (vs. placebo)
MAP (mmHg)	VTWG group (*n*=30)	102.37 ± 5.38	96.57 ± 6.55	-5.80 ± 4.44	<0.0001	<0.0001	6.77 [4.21, 9.33]	<0.0001
	ETRG group (*n*=30)	102.07 ± 5.59	95.03 ± 6.82	-7.03 ± 5.44	<0.0001	<0.0001	7.90 [5.14, 10.67]	<0.0001
	Placebo group (*n*=29)	100.00 ± 4.94	101.38 ± 6.19	1.38 ± 5.19	0.163	—	—	—
Pulse rate (beats/min)	VTWG group (*n*=30)	82.60 ± 10.80	78.30 ± 10.16	-4.30 ± 11.60	0.052	0.669	1.48 [-3.87, 6.84]	0.582
	ETRG group (*n*=30)	76.60 ± 11.40	73.20 ± 9.32	-3.40 ± 8.03	0.028	0.419	3.15 [-1.97, 8.27]	0.223
	Placebo group (*n*=29)	87.14 ± 11.30	81.48 ± 11.40	-5.66 ± 12.64	0.023	—	—	—
hs-CRP (mg/L)	VTWG group (*n*=30)	1.06 ± 1.01	1.05 ± 0.97	-0.01 ± 0.85	0.956	0.760	-0.13 [-0.54, 0.28]	0.537
	ETRG group (*n*=30)	0.90 ± 0.72	0.94 ± 0.73	0.04 ± 0.70	0.766	0.591	-0.09 [-0.47, 0.29]	0.634
	Placebo group (*n*=29)	0.96 ± 1.00	0.87 ± 0.87	-0.08 ± 1.01	0.660	—	—	—
Nitric oxide (μmol/L)	VTWG group (*n*=30)	50.71 ± 34.09	54.29 ± 38.99	3.58 ± 45.76	0.671	0.836	-12.78 [-29.50, 3.93]	0.131
	ETRG group (*n*=30)	50.59 ± 19.60	56.57 ± 24.68	5.98 ± 29.51	0.276	0.531	-16.28 [-29.14, -3.42]	0.014
	Placebo group (*n*=29)	36.48 ± 13.25	38.10 ± 20.25	1.62 ± 23.12	0.708	—	—	—

Values are presented as mean ± SD; (1) Within-group differences between baseline and week 12 were analyzed using a paired t-test; (2) Between-group differences in changes from baseline were analyzed using an independent t-test for VTWG vs. placebo and ETRG vs. placebo, respectively; (3) Adjusted mean differences (95% CI) and p-values were obtained using ANCOVA adjusted for the baseline value of each outcome, with differences calculated as placebo minus intervention. Abbreviations: MAP, mean arterial pressure; hs-CRP, high-sensitivity C-reactive protein; NO, nitric oxide; ANCOVA, analysis of covariance; CI, confidence interval.
